# Use of virtual reality simulation in preparation for practical training in healthcare education – a mixed method study with students and educators

**DOI:** 10.1186/s12909-026-09579-9

**Published:** 2026-05-30

**Authors:** Nina Skjaeret-Maroni, Siri Merete Braendvik, Ida Juberg, Sissel Horghagen, Ann-Kristin Gunnes Elvrum

**Affiliations:** 1 Department of Neuromedicine and Movement Science, Faculty of Medicine and Health Science, Norwegian University of Science and Technology (NTNU), Trondheim, 7030 Norway; 2 Clinic of Rehabilitation, St. Olav’s Hospital, Trondheim University Hospital, Trondheim, 7006 Norway

**Keywords:** Virtual Reality, Simulation-based education, Active learning, Communication skills, Healthcare education, Healthcare discipline education

## Abstract

**Introduction:**

Simulation-based education promotes active learning and reflective practice in healthcare education. With the advancement of immersive technologies, virtual reality (VR) enables simulation of complex clinical interactions in a safe and engaging environment.

**Purpose:**

This study explored the experiences and perceptions of students and educators regarding the use of VR-based dialogue simulations as preparation for practical training in physiotherapy and occupational therapy education.

**Methods:**

A concurrent mixed-methods design was applied. Ninety-three second-year bachelor students participated in two short VR scenarios simulating communication with a child with cerebral palsy and her father. Data was collected through a post-simulation questionnaire for the students (*n* = 49) and a focus group interview with five educators. Questionnaires were analyzed descriptively, while reflexive thematic analysis was employed on the interviews.

**Results:**

Students described VR simulation as a valuable and safe learning experience. They highlighted the importance of observing peers, engaging in structured reflection, and receiving feedback. The VR component contributed by providing brief but immersive encounters with the child and parent avatars, which enriched the subsequent reflections. Educators emphasized that the simulations triggered emotional engagement and promoted deep learning, particularly during the debriefing phase. Both explicit learning outcomes (e.g., practicing open-ended questions) and implicit outcomes (e.g., increased confidence, emotional resilience) were identified. Technical support and a structured pedagogical model were essential for successful implementation.

**Conclusion:**

VR-based simulations represent a promising pedagogical approach for developing communication skills in healthcare education. Their effectiveness depends on constructive alignment of learning objectives, structured facilitation, and reliable technical support. Future research should examine long-term skill transfer and optimize VR design for complex interpersonal interactions.

**Supplementary Information:**

The online version contains supplementary material available at 10.1186/s12909-026-09579-9.

## Introduction

Developing autonomous and reflective thinking is vital in the education of health professionals [1]. To support this, integrating Evidence-Based Health Care (EBHC) principles into healthcare curricula is widely recommended [2]. Interactive teaching methods that combine clinical practice with learning are considered the most effective for teaching EBHC, outperforming traditional lectures or isolated instruction [3, 4]. Active learning is widely recognized for enabling students to engage in authentic tasks that foster critical thinking, collaboration, and accountability [5–7]. Although the concept lacks a universal definition [8] and is used in a wide range of concepts [9], it generally refers to teaching practices that involve students actively engaging in the learning process [10]. Carefully designed active learning activities can enhance students’ ability to integrate new information into their existing knowledge base. These activities should draw on a well-constructed knowledge foundation and target higher-order learning objectives such as application, analysis, and evaluation. Incorporating diverse methods for encoding the learning task further strengthens this process [11, 12]. To achieve these outcomes, teaching strategies should be aligned with intended learning objectives and assessment tasks, a principle known as constructive alignment [13]. This approach ensures that students engage in activities that directly support the desired competencies, making active learning particularly relevant in health education.

Simulation is a student active learning method involving activities that enable students to acquire deeper understanding, competence, and capacity for clinical reasoning and conscious reflection [14]. From a pedagogical perspective, simulation aims to foster transformative learning. This type of learning involves changing one’s frame of reference and is facilitated through critical reflections [15]. Simulation scenarios mimic real life clinical scenarios, thus representing a context where students can explore various aspects of a practical skill, both technical and non-technical [16, 17]. When using simulation as a learning method for students, it is recommended to follow a set structure facilitated by a qualified educator: I) prebriefing, which involves preparing the student for the simulation session; 2) scenario execution, which should align with the objectives and learning outcomes of the simulation; and 3) debriefing, where the simulation experience is reflected upon [18]. The debriefing section includes both feedback and feedforward, where critical reflection provides formative feedback in which the students can reflect on the scenario and how experiences from participation can be transferred into real life practice [19]. Learning is enhanced through the exchange of feedback and feedforward among peer students and between students and the facilitator. As described by Hattie and Timperley [20], feedback addresses the question “Where am I now?” while feedforward focuses on “Where do I go from here?”. In addition, guided reflection may encourage students to explore critical elements of their experience to help them connect theory, practice, and research [14]. Simulation-based training offers a safe, standardized environment for practicing responses to rare or critical events without risking patient safety [21].

Advances in technology have accelerated the integration of Virtual Reality (VR) into simulation-based education. VR simulators are increasingly used in both clinical training and academic programs, offering immersive, repeatable, and highly interactive learning experiences [22, 23]. While simulation-based education provides a pedagogical structure built around prebriefing, scenario execution, and guided debriefing, VR represents a distinct modality within this framework. The pedagogical benefits typically associated with simulation, such as psychological safety, feedback, and structured reflection, derive from the simulation design rather than the technology itself [14, 24]. In contrast, VR contributes specific technological affordances, including a heightened sense of presence, immersion, and embodied interaction [25], which can influence learners’ emotional engagement and perspective-taking in ways that traditional role-play cannot easily replicate [26]. Additionally, gamification techniques, which incorporate game-like elements into learning, enhance engagement and motivation. By encouraging active participation, these methods support deeper learning and behavioral change [27–29]. The use of VR in simulation allows students to act and interact in real-time, promoting experiences and feelings within the computer-generated, realistic or artificial three-dimensional (3D) environment [22, 23]. Studies have suggested that using VR may enhance knowledge and skills among health professional students compared to traditional or other digital learning methods [30]. More interactive VR experiences have been shown to be more effective than less interactive ones in healthcare simulation [31]. Studies indicate that higher levels of interactivity in VR environments enhance learner engagement, knowledge retention, and skill acquisition. For example, immersive VR simulations that allow users to manipulate virtual tools or make clinical decisions in real time have been associated with improved performance and deeper learning compared to passive VR experiences [32, 33]. This is likely due to the active learning principles embedded in interactive VR, which promote cognitive engagement and experiential learning [34].

However, the possibilities of VR are vast, and evidence regarding use of VR to practice non-technical skills such as communication in challenging clinical situations remains limited, highlighting the need for further research into the perceived impact of interactive forms of VR. Furthermore, to support effective student learning utilizing technology, the literature emphasizes the importance of continuous digital skills development among educators, as well as access to high‑quality digital learning resources [35]. Collaborative development and implementation of simulation activities across educational programs may have the potential to promote students’ communication skills while simultaneously supporting interdisciplinary collaboration and perceived professional development among educators.

Hence, this study aimed to explore the experiences and perceptions of students and educators regarding the use of VR-based simulations as preparation for practical training in higher healthcare education. The study examined students’ learning outcomes, the challenges faced during implementation, and the future possibilities of this pedagogical method, thereby informing the collaborative development of VR dialogue simulations that can enhance communication skills and digital competence across professional boundaries.

## Methods

### Design

A concurrent mixed-method design was chosen because it enabled capture of complementary dimensions of the learning experience and capture of different aspects of the research question [36, 37]. A questionnaire was used to collect immediate, individually anchored responses regarding students’ perceptions of safety, usefulness, and mastery. This approach enabled the inclusion of a larger proportion of participants and captured a broader range of experiences. The anonymous format was intended to support candid reflections, particularly regarding potentially sensitive aspects of simulation such as vulnerability and performance, which may be influenced by group dynamics in interviews or focus groups. The questionnaire was administered directly after the VR simulation to ensure timely insights into students’ experiences before they transitioned into internships or other educational activities. The questionnaire included both closed-ended and open-ended free-text questions, allowing for both structured data and richer qualitative input. To complement the student data, a semi-structured focus group interview was conducted with participating educators who facilitated the VR simulation to encourage dynamic discussion and allow participants to build on each other’s observations [38]. The focus group interview allowed the educators to articulate how the VR scenarios shaped group dynamics, emotional engagement, and reflective dialogue, in addition to interdisciplinarity and own professional development.

### Context

**Fig. 1:**
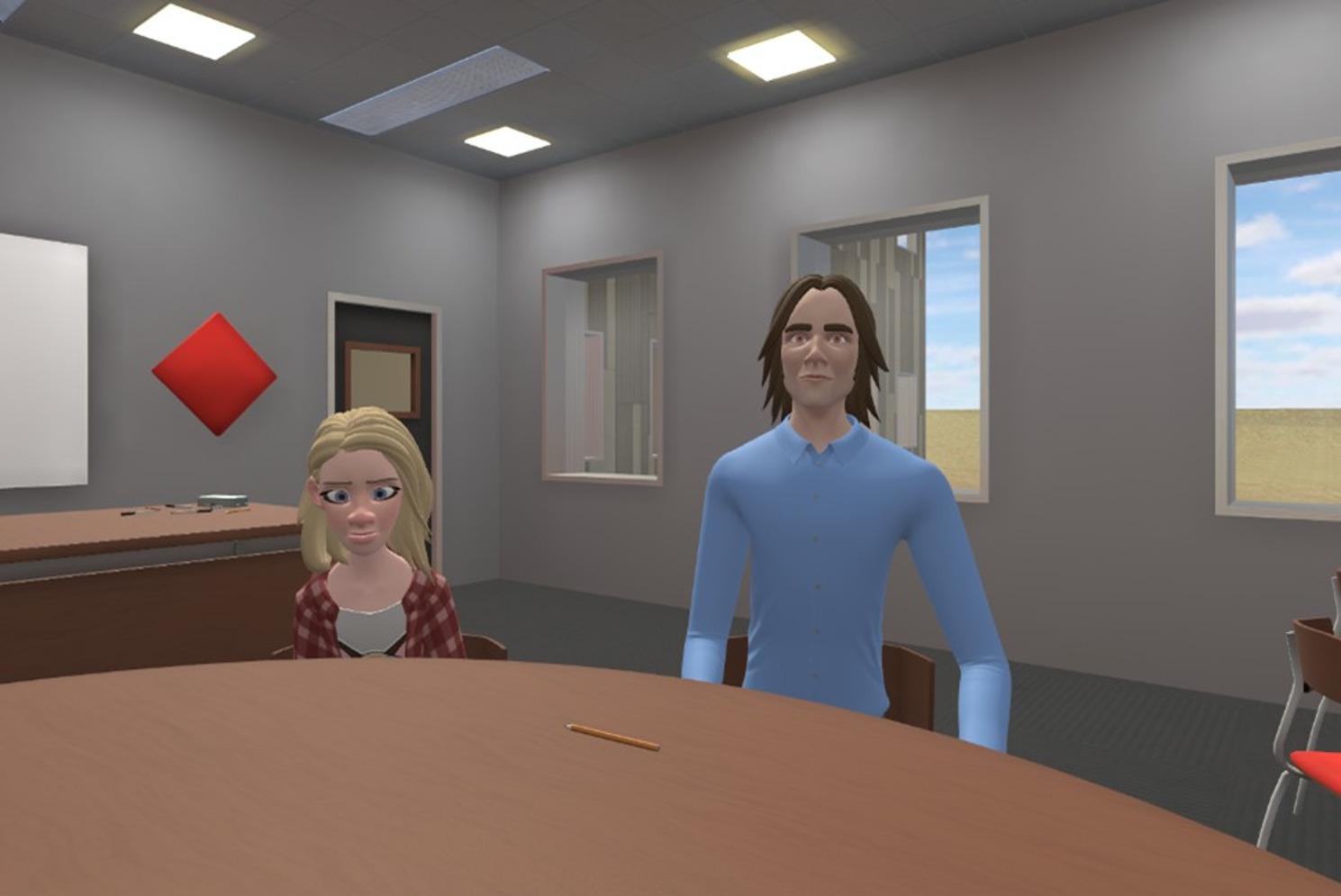
Screenshot from the VR room during the dialouge with Emma and Petter

The interactive VR-simulations described in this study were developed through a collaborative project between the physiotherapy and occupational therapy programs at the Norwegian University of Science and Technology, Trondheim, Norway. Two animated VR-based scenarios were developed in collaboration with Fynd Reality AS, Hamar, Norway. The scenarios are based on a mapping dialogue with 10-year-old Emma, who has cerebral palsy, and her dad, Petter, where students are asked to identify which activities Emma struggles performing in her daily life (Fig. 1). The simulation dialogues have preset answers and are controlled by a facilitator. In the first scenario, “Silent Emma”, the students meet a quiet and shy girl who is not willing to share much information, and where her dad, Petter, is trying to encourage her to say something herself. In the second scenario, “Dominating Dad”, the students meet a frustrated and dominating dad who answers all the questions for his daughter without giving her time or opportunity to answer herself. Both scenarios last approximately 1.5 min.

The VR dialogue scenarios were delivered through the CORE platform (Fynd Reality, Norway), operated from a PC connected to an HTC Reverb VR headset. Students interacted with virtual characters using natural voice communication only. The avatars’ responses and the direction of the dialogue were controlled in real time by the facilitator, who selected among predefined response options within the CORE interface to ensure consistent scenario flow.

The trialing of the VR-scenarios was based on full scale simulation methodology, rooted in specific learning outcomes for the students. In this case, the learning outcomes covered fundamental health education communication skills, focusing on asking open-ended questions within a family-centered perspective. A total of seven educators from the physiotherapy and occupational therapy programs were involved as facilitators for two or more student groups, including the first (NM) and the last (AKGE) author of this paper. As facilitators, the educators were responsible for conducting the prebriefing, guiding students through the simulation, and leading the debriefing sessions. During the simulation, facilitators did not interact directly with the students in the dialogue but controlled the progression of the VR scenario by selecting predefined response options. Six of the educators had completed the basic training required for facilitator certification. One educator received written and oral information and observed another educator facilitating the VR-dialogue simulation and group reflections before she acted as a facilitator. Prior to the simulation, a guideline was developed and distributed to all facilitators. This guide outlined the intended learning outcomes, technical setup, scenario sequence, and example questions, thereby ensuring that all groups experienced an equivalent prebriefing, simulation process, and debriefing structure.

Before the VR-simulation, the students attended a pre-lecture where rules, expectations, and learning outcomes were explained. To standardize implementation across all student groups, the same introductory explanation about simulation structure and expectations was provided. Furthermore, the students were introduced to the VR-simulation environment and informed about the subsequent debriefing and reflective activities. Students were subsequently divided into groups containing well-known co-students. During simulation, the students had different roles in the group, either being the one conducting the dialogue simulation or being a respondent listening to the others and responding based on feedback and feedforward principles. Depending on the group size, each student completed one or both scenarios. All students in a group were seated together in the same room along with the facilitator, where they could see and hear the simulating student and the responses from the avatars, Emma and Petter. After each simulation, a debriefing session focusing on feedback and feedforward was carried out.

### Participants

All second-year bachelor students in physical therapy (*N* = 61) participated in the first test of the VR-dialogue simulation as part of their compulsory preparation before their first period of supervised professional training. Additionally, second year bachelor students in occupational therapy were invited to voluntarily sign up for testing of the VR-dialogue simulation as part of their education, and 32 students took part in the testing. The students conducted the simulation in groups of 4–8 students. The physical therapy students conducted the testing with their regular study group. For the occupational therapy students, two or more regular groups were merged as participation was voluntary. Physiotherapy and occupational therapy students were included because both programs share core communication-learning outcomes related to family‑centered practice and conduct their clinical training within similar community and hospital contexts. The VR scenarios were co-developed by both programs and implemented as a shared teaching activity. Including both student groups enabled exploration regarding whether a common simulation design supported learning across two professional educations with overlapping competence requirements. Although comparing responses between programs was not a primary analytical aim, the mixed-methods design allowed us to examine whether experiences differed or aligned across the two groups.

For the focus group interview, five educators who had not been involved in the development of VR-simulation dialogues were recruited to share their experiences regarding facilitation of the VR-simulation. Three were educators at the physiotherapy program, and two at the occupational therapy program. The educators had varying levels of teaching experience and prior exposure to simulation-based teaching.

### Data collection

Following the simulation, a self/developed, anonymous digital questionnaire was distributed to the students, consisting of 16 closed-ended questions rated on a 5-point scale ranging from “strongly disagree/to a very small extent” to “strongly agree/to a very large extent”. Additionally, two open-ended questions invited students to describe up to three positive aspects of their experience and three areas for improvement regarding the simulation, providing them with an opportunity to elaborate on their grading responses (see supplementary file for questions). The questions addressed their impression of simulation as a learning method, the relevance of the learning outcomes to the simulation, their experiences of acquiring the roles as simulating and respondent, the reflections in the debrief sessions, and their perception of safety and mastery.

Prior to the focus group interview, the authors of this study collaboratively developed an interview guide comprising open-ended questions tailored to structure the discussion and encourage reflective dialogue, allowing the educators to articulate and interpret their perceptions of the VR simulation in the context of their teaching practice. The term “educator” is used when referring to participants in the focus group interview, while “facilitator” refers to the role these same individuals assumed during the simulation sessions. The interview guide was based on topics such as (1) practical implementation of the VR simulation, (2) students’ benefits from the VR simulation, (3) collaboration between the physical therapy and occupational therapy programs, and (4) relevant experiences from the simulations (see supplementary file for full interview guide). The focus group interview was moderated by the second author (SMB) and lasted approximately 90 min. It was digitally audio recorded using the encrypted Dictaphone app provided by Nettskjema [39]. Two of the authors were assistant moderators (NSM and SH) and took notes to capture impressions that emerged during the discussions. The recorded transcripts were transcribed manually verbatim and anonymized for analysis.

### Data analysis

The questionnaires were downloaded into a joint Excel-file and descriptively analyzed. Data was first checked for completeness and consistency for the two bachelor programs separately. Categorical variables (e.g., bachelor program and number of replies) were summarized using frequencies and percentages. Further, responses from the open-ended questions were linked. Data were visualized using bar charts to illustrate distributions and trends where appropriate.

A reflexive thematic analysis was conducted on the focus group transcript to identify key patterns of meaning within the data [40]. This flexible method is suitable across theoretical frameworks and enables a nuanced exploration of the participant’s subjective experiences and interpretations [40]. The analysis was inductive, semantic, and realist in orientation, allowing the data to guide the coding and development of themes, following Braun and Clarke’s six- phase approach [41]. In the first phase, the first and last author (NSM and AKGE) independently read and re-read the transcript to become familiar with the data, noting initial impressions, reflections, and questions. They then met to discuss these impressions, paying particular attention to similarities and differences in their interpretations. Given their prior involvement in developing and facilitating the VR simulation, both authors engaged in explicit reflexive discussions to acknowledge and critically consider how their pre-understanding and experiences might influence the analysis. This process aimed to support a more inductive and data-driven approach while recognizing the authors’ active role in constructing meaning from the data. The last author documented key points from these discussions and noted initial codes, which were subsequently discussed. In the second phase, the last author re-read the transcript and systematically coded meaningful segments of the data using an inductive approach. In the third phase, these codes were examined to identify patterns, similarities, and connections forming the basis for initial themes. In phase four, preliminary themes were reviewed for coherence in relation to both the coded data and the transcript, as well as their relevance to the research objective. To increase rigor, the first and last authors conducted this review independently before meeting to compare, discuss, and collaboratively revise the themes to reach consensus. This iterative process involved jointly refining the themes by merging, splitting, or discarding preliminary themes as needed. It then progressed into the fifth phase, in which both authors worked together to name and clearly define the themes to ensure they captured their essence and analytic focus. In the sixth phase, the last author produced a draft of the final report presenting the themes in logical order and supporting them by illustrative quotes from the participants. During this phase, all authors engaged in iterative discussions to refine interpretations and discuss their implications for teaching practices, challenges, and future implications. This collaborative process was particularly important, as two of the authors (SB and SH) had not been involved in the development and facilitation of the VR simulations and therefore contributed to a more independent perspective. Reflexivity was considered throughout the analytic process. The authors continuously reflected on their perspectives and potential influence on the analysis, contributing to transparency and methodological rigor.

#### Analytical lens

Based on general principles of simulation-based education, the tenets of the debriefing process was used as an analytical framework to interpret our findings. Debriefing is recognized as a core component of simulation-based education, assisting in the development of clinical reasoning and reflective thinking [24]. Three key elements are considered essential to facilitate the pedagogical potential of debriefing: (i) structured models in which learning outcomes guide debriefing and reflections; (ii) a trusting and supportive learning environment in which students feel free to share experiences; and (iii) the facilitator’s role in the process using feedback, debriefing, and guided reflection [14, 24]. Feedback is provided as unidirectional exchange of information intended to enhance understanding or performance, while debriefing is a bidirectional dialogue that fosters reflective thinking during and after the simulation. Guided reflection is used to encourage learners to explore the critical elements of an experience to gain insight, helping them connect theory, practice, and research [14]. During the debriefing process, facilitators maintain a safe learning environment while they observe the behavior of the students, encourage open discussion, provide appropriate feedback, facilitate reflective thinking and generate solutions to unanticipated situations using feedforward and guided reflections [14]. In our analysis, we used the framework for the debriefing process to interpret how facilitators supported student growth while managing the dynamic and sometimes unpredictable nature of simulation-based learning. In line with simulation-based education standards, we assumed psychological safety as a prerequisite for effective debriefing, group reflection, and facilitator–student interaction. Because all student data were anonymous, we could not explore how individual factors or implicit biases might have influenced psychological safety or interactional patterns during the simulation.

### Ethics

The online questionnaire was submitted to The Norwegian Agency for Shared Services in Education and Research (SIKT) for assessment. As no identifiable or potentially identifiable personal data were collected, SIKT determined that the project did not require ethics approval in accordance with Norwegian research ethics and data protection regulations for anonymous data collection. As the survey was fully anonymous, informed consent was obtained implicitly through voluntary completion of the questionnaire. For the focus group interview, approval was obtained by SIKT (ref.nr 654208) prior to study commencement. All participants in the interview received written and oral information about the study as well as their right to withdraw from the study at any time without giving a reason. Informed, voluntary written consent was collected prior to the interview. The study was conducted in accordance with the Declaration of Helsinki.

## Results

### Student replies

Of the 93 students participating in the simulation, 49 (46%) replied to the questionnaire. Of these, 14 students chose not to answer the last two open-ended questions. Of the students who responded, 33 (67%) were studying physical therapy and 16 (33%) were studying occupational therapy.

#### Students liked simulation as a learning method

**Fig. 2:**
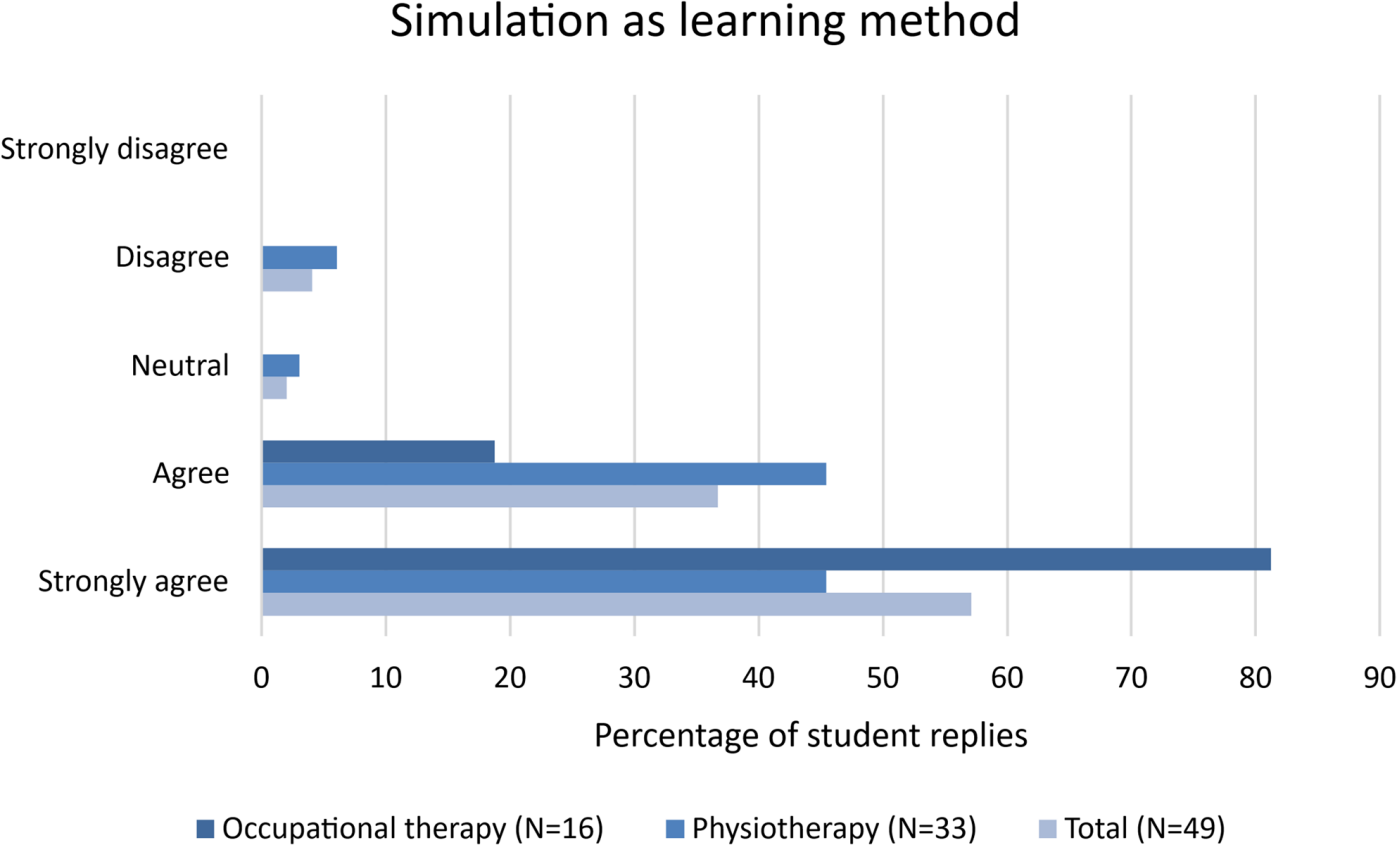
Student perceptions of VR simulation as a learning experience

Forty-six (94%) of the students stated that they liked simulation as a learning method to a large or very large extent. The remaining three students were neutral or disagreed with the statement (see Fig. 2) and elaborated that they felt high levels of stress and discomfort during the simulation. Forty-one (84%) of the students stated that they felt safe doing simulation in the groups, while six were neutral and two disagreed. Thirty-five (71%) further stated that they were left with a sense of accomplishment after the simulation and subsequent debrief session. Here ten were neutral, three disagreed, and one strongly disagreed. The fact that the groups were small and familiar was important for the students to help them create a safe environment. Several stated that being vulnerable in front of the rest of the group and observing a fellow student being vulnerable helped them to challenge themselves in a somewhat uncomfortable situation. One student stated, “*I got to feel in my body what it’s like to end up in an uncomfortable situation*”. A few students who did not feel safe or accomplished noted they wanted more information beforehand and found the dialogues too short to practice communication skills. Interestingly, students generally linked feelings of safety, opportunities to observe peers, and perceived mastery to the simulation structure (small groups, facilitation, and debriefing), rather than to the VR technology itself.

All occupational therapy students felt that the simulation was a valuable learning experience to develop their communicative skills and meet the learning outcomes. One of the students pointed out that “*you learn how to involve everyone in the conversation*”. For the physiotherapy students, 28 (85%) felt like the simulation gave them the opportunity to practice their communicative skills, while the last five did not agree. Several of the students stated that the dialogues were too short to practice communicative skills. A few of the students also pointed out that the utilized simulation technology had not progressed enough to be used as a tool for improving communication skills, as the avatars’ responses did not always address the questions that were asked. One of the students even stated that using an animated simulation resulted in lower quality conversations. At the same time, some students emphasized that the VR‑specific contribution with the use of an immersive headset and avatar‑based interaction made the scenario feel different from classroom role‑play and increased emotional engagement, even though the conversations were brief.

#### Experiences from the reflections

**Fig. 3:**
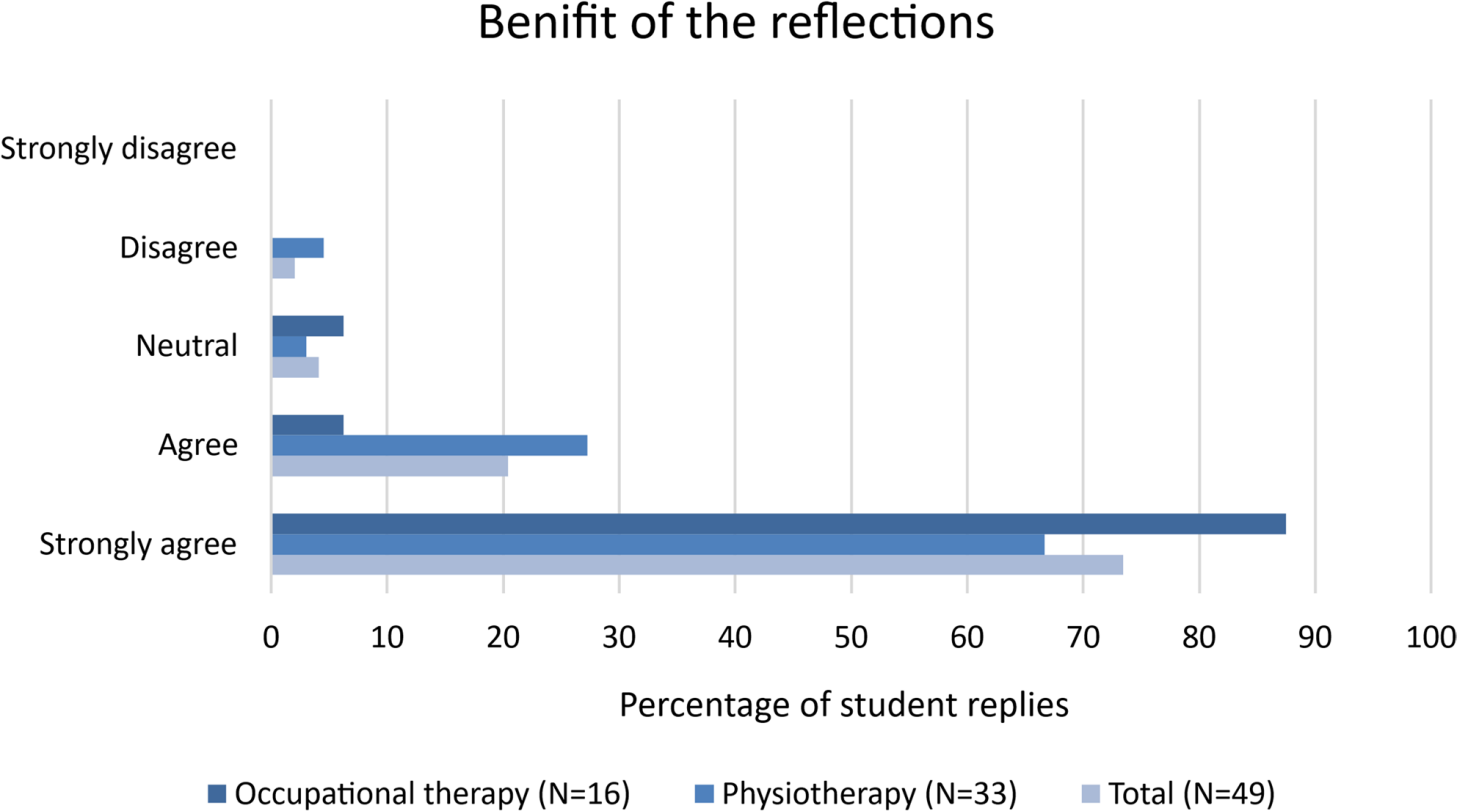
Student perceptions of learning from reflections

Forty-one (84%) of the students stated that being the one conducting the simulation provided them with academic benefit. However, eight students (16%) did not feel like they gained any learning experiences from performing the simulation (see Fig. 3). On the other hand, all 49 students stated that being respondents provided them with academic benefit. Several of the students highlighted that they appreciated being part of a group where everyone completed the VR simulation, so that they got to observe how fellow students solved the task. One student stated, “you get the opportunity to observe a peer being vulnerable”, as a positive outcome from the simulation.

Having the opportunity to reflect upon the dialogue afterwards in the group was the aspect of the simulation that the students provided most comments on in the questionnaire. Thirty-one (94%) of the physiotherapy students and 15 (94%) of the occupational therapy students agreed or strongly agreed that the reflections afterwards provided them with learning benefits and that they learned from each other during the discussions. While two answered neutrally, one student disagreed that the reflections and discussions afterwards provided learning benefits. However, these students did not elaborate on their reasoning in the open-ended questions. Some of the students that found the reflection beneficial appreciated having plenty of time to discuss and reflect together with the group and the educator, where they got to highlight which strategies worked well and how they could use these further in other settings.

### Educators’ experiences

In our reflexive thematic analysis, three themes were identified that reflect educators’ experiences using VR-based simulations to prepare students for practical training in higher healthcare education. The first theme highlights that the educators found VR-based simulations to be an effective tool for promoting student reflections and learning. The second theme describes how the educators perceived technical support and a structured pedagogical outline as crucial for building confidence in facilitating VR-based simulations. The third theme captures the perceived benefits of this pedagogical method for the educators themselves. The debriefing process framework is used to interpret each themes’ findings.

#### Effective tool for promoting student reflections and learning

Factors contributing to promoting student reflections and learning by using VR-simulation are elements that the educators highlighted using descriptions such as “*more real*”, “*triggering emotions*”, “*reflections*”, “*learning*”, “*sense of security*”, “*practice*”, “*acceptance*”, “*clear expectations*”, and “*outcomes*”.

All five educators expressed that the VR simulations were realistic and helped students immerse themselves in the scenarios. As educator 1 said,” It’s *more real than if one of us plays a mother or a father”*. The educators considered that it was better than role playing because the students forgot about the others in the room once they put on the VR-glasses. In addition, emotions were more easily triggered. Educator 2 talked about how this created engagement, especially when the students encountered Peter as a dominant father:

… *we have quiet Emma who doesn’t say a thing*,* and then you have the dominant father who won’t shut up. No matter how much you tell him to be quiet*,* he takes over. And that triggers varying emotions in the students. So*,* I think that having two scenarios at each end of the scale does a lot for the students’ reflections and learning.*

The educators appreciated that the VR simulation allowed the students to practice communication in a safe setting. As Educator 5 explained, interacting with a child and parent in a “*somewhat harmless way*,* not in a real setting”* helped initiate important learning processes. Educator 2 added that students gained confidence by observing and reflecting on each other’s varied approaches: *“They learned a lot from observing that we do it so differently*,* yet still come to similar conclusions*,* there are so many ways to do it*,* it doesn’t have to be perfect*”. The educators also noted that even brief one-and-a-half-minute VR simulations sparked rich reflections and meaningful discussions among students, contributing to valuable peer-to-peer learning. They emphasized that the most significant outcome of the VR experience lay not in the simulation itself, but in the depth of engagement, reflections, and learning that followed in the debriefing sessions.

Group size also played a key role in fostering reflective dialogue. Educator 3 noted that having four to six students in a group enhanced the quality of reflections compared to working in pairs, as more participants meant more perspectives and observations to draw from. In addition, the educators emphasized the importance of a supportive group environment where each student feels genuinely seen and heard by both peers and the educator. Several highlighted how the acceptance from the rest of the group contributed to a sense of safety and mastery, even when things did not go as planned, as described by Educator 4:

*They also experienced acceptance from the group*,* when it didn’t work*,* if they didn’t manage to ask open questions*,* or didn’t manage to achieve the learning objectives*,* they still felt some form of mastery*,* because they had a fellow student who could sit and say that in the same situation*,* he would probably also been ‘caught in that trap’.*

The educators highly valued the clarity of the student learning outcomes, focusing on asking open-ended questions and acknowledging Emma and her father. They emphasized that having a few clear learning outcomes that the students could master was crucial for achieving the desired outcomes. The educators also reflected on how the students gained more from the VR simulations than just the explicit learning outcomes. They developed the courage to engage in unfamiliar situations and the ability to give and receive feedback, skills not formally stated in the objectives. Educator 5 referred to these as “*hidden*” learning outcomes, noting that: “*There are actually some hidden learning outcomes*,* like having the courage to do this*,* because it’s a new challenging situation. You have to tolerate receiving and giving feedback to yourself and others. But we don’t really say that.”* The educators agreed that these implicit learning outcomes were best left “hidden” before the VR simulation. Rather than highlighting them in advance, they believed it was more effective to let such outcomes emerge naturally through the simulation design and the reflective discussions that followed.

This theme’s findings indicate that the most meaningful learning occurred during the reflective process following the simulation. Even brief simulations sparked rich peer discussions, with the group format enhancing reflections and shared learning. This aligns with established principles of debriefing, including feedback, reflective dialogue, and guided reflection [14]. Facilitators played a key role in creating a psychologically safe environment, where group acceptance and non-judgmental feedback fostered a sense of mastery, even when students struggled to meet learning objectives. This highlights the facilitator’s role in balancing challenge with support, ensuring that the debriefing process is constructive and that students feel seen and heard. While clear objectives, such as asking open-ended questions, provided structure for feedback and reflection, deeper learning emerged through guided reflection.

#### Technical support and a structured pedagogical outline created confidence

The importance of technical support from others and a structured teaching model was highlighted by the educators to avoid “*anxiety*”, “*stress*”, and “*fumbling*”, and to create “*control*”.

During the VR simulation, the educators expressed that it was crucial to have access to good technical support from a *“super-user”*, as expressed by Educator 1: *“It was important that others knew the system better than I did … so things went according to plan. And if there were any small issues*,* they got resolved”*. This created a sense of security. Additionally, several educators suggested having an instructional video, or a *“crisis book”* to be able to handle minor issues independently. They agreed that technical failures would have caused a lot of stress, and potentially undermined the entire experience, something many were concerned about in advance. As Educator 3 stated, “*It either works*,* or it doesn’t*,” describing the irrational anxiety she felt despite thorough preparation. In addition to technical support, good logistics and a clear schedule were seen as vital. Educator 4 highlighted the value of having one person with full oversight of the setup, ensuring that everything was well organized. This contributed to the success of the simulation and increased motivation to use VR in the future.

A well-structured pedagogical model with a written outline also played a key role in enabling educators to act as facilitators, even without formal training. The educators noted that the outline clarified their responsibilities, made them more confident, and helped them to promote a trusting learning environment, making the students feel more secure in an otherwise unfamiliar setting. As Educator 2 explained, the structure allowed *“… students to speak freely*,* while still being able to maintain the flow of the conversation”*. Educator 4 described how the setup helped students feel prepared, despite the vulnerability of being observed by peers:

*A bunch of students coming in and … being a bit ‘out there.’ They are in a way going on stage. They’re going to be visible to their peers*,* and ensuring their sense of security is important*,* even though you yourself might feel like you’re fumbling a bit. And that … the support provided through the simulation*,* it seemed like with this setup*,* the students were well prepared.*

Educator 5 supported this, emphasizing the value of preparation and prebriefing: *“… the fact that things are well-prepared and that they know what they will somewhat encounter*,* even if they don’t know everything (…) I think that is very important”.*

This theme illustrates how reliable technical support and clear logistics during VR-simulations reduced stress and created a sense of control for the educators, enabling them to ensure that students felt feel free to share their experience in a psychologically safe environment, aligning with the second key element of debriefing: establishing a trusting and supportive learning environment [24]. In addition, the structured pedagogical model enabled the facilitators to create conditions for meaningful reflection and peer learning, core aspects of effective debriefing. These findings underscore the value of planning and outlining the debriefing process beforehand to support the facilitator in their role when guiding the debriefing process [24, 42].

#### Experienced benefits for the educators

This theme highlights the educator’s perceived benefits of this pedagogical method for themselves as educators. Words such as “*learned*”, “*fun*”, “*enrichin*g”, and “s*trengthened*” were used to describe their increased sense of mastery as well as experienced gains of collaborating as facilitators across the educational programs.

The educators described the experience of facilitating the VR-simulations with student groups from both educational programs as highly valuable. They emphasized how much they appreciated listening to students’ reflections and observing how students adapted their communication strategies after their performance. This process allowed educators to witness students’ growth and learning. Educator 4 described how she found it incredibly enriching to observe how the students “*gradually gained the freedom to dare and try*,* where nothing was considered wrong. I think that’s when we really make progress—when they manage not to limit themselves but instead explore together.”* Similarly, Educator 2 said:

*… we were literally just sitting there*,* like*,* ‘Yeah*,* what do you think? And why?’ It was them who were talking and them who were learning. We didn’t have to sit there and—it was not us who were telling. I think so much important stuff happens in exactly this kind of reflection. And also in this feedback-feedforward situation.*

By facilitating student reflections through the debriefing process, the educators expressed that they gained a sense of mastery as educators. Rather than leading the discussion, they guided students through feedback and feedforward, which they felt significantly strengthened their role. As Educator 3 noted, she now feels like *“a more confident mentor in a guidance situation”*. The educators agreed that the facilitation course had strengthened their mentoring skills and reflective practices. Educator 4 explained:

*I think my approach would have been different if I hadn’t taken the facilitation course. And perhaps I wouldn’t have been so focused on the mastery aspect and how I structure the reflection afterward. So*,* to put it mildly*,* I think my role has been significantly strengthened*.

Furthermore, the educators highlighted the value of learning from each other during planning, breaks, and post-simulation discussions. The social and interdisciplinary aspects of the collaboration were particularly appreciated. Educator 4 shared how incredibly fun she thought it was “*to work with colleagues both across and within disciplines - not because we necessarily come from different professions*,* but because we get to know each other and it was a social arena for us as well.”* In addition, the educators viewed this collaboration across disciplines as an effective use of resources. Educator 3 remarked: *“we utilize the resources*,* because as you say*,* not everyone has the facilitator course*,* not everyone might need to have it*,* and then we can rather join our forces*”.

While acknowledging that simulation requires significant teaching resources, the educators agreed that the student’s engagement and learning far outweighed those of traditional lectures. Educator 1 noted: *“In much of the teaching*,* not everyone gets to say something. But here*,* everyone gets to speak—that’s something I liked and want to change”.* They discussed the potential of reducing some of the traditional lectures and replacing them with increased student self-study of theoretical curriculum. This shift could enable the allocation of more time and resources to student-active, group-based learning. Educator 5 shared an example of how she and a colleague had incorporated feedback and feedforward into other learning activities: *“We actually extracted those elements from the VR simulation into an activity at the occupational therapy education a week or two afterwards*,* where they used it in the groups”.* This inspired further reflection on how such pedagogical elements could be applied more broadly across educational contexts. Despite the effort required, the educators agreed that this approach should be prioritized. They further discussed ways to optimize resource use, such as forming a dedicated interdisciplinary team to develop ready-to-use educational resources.

This theme describes the benefits the educators experienced from contributing as facilitators. Using the debriefing framework as an analytical lens reveals how the educators supported student learning through structured reflection, a safe learning environment, and skilled facilitation [14, 24]. At the same time, the experience contributed to educators’ professional growth and inspired broader pedagogical innovation. Educators reported a shift in their role, that enhanced their own sense of professional mastery. In addition, they appreciated learning from colleagues and saw the potential for resource sharing and scaling simulation-based methods across educational programs.

## Discussion

This study suggests that VR-based simulation is perceived as a valuable pedagogical method for preparing healthcare students for practical training. The majority of students reported feeling safe during the simulation and experienced learning benefits both as active participants and as observers. The reflection phase following the simulation was particularly emphasized as a key source of insight and professional development. Educators viewed the VR simulations as an effective tool for promoting student reflection and learning, and they highlighted the importance of technical support and a structured pedagogical model to ensure quality implementation. Additionally, educators reported a sense of professional growth and benefit through interdisciplinary collaboration and facilitation. The student and educator data showed clear convergence in emphasizing reflection and debriefing as central to the VR learning process, while also offering complementary perspectives on the simulation experience. Students focused on emotional engagement and peer dynamics, whereas educators emphasized the benefits of a clearly structured pedagogical approach supported by facilitation methodologies. Some divergence was also evident in perceptions of the VR technology, particularly regarding its added value, where students expressed more varied and sometimes critical views, while educators more consistently framed VR as a useful tool for engagement and reflection. Together, these findings provide a more nuanced understanding of how VR-based simulation supports learning within a structured educational framework.

### VR simulation as an active learning strategy

The findings suggest that participants perceived VR-based simulations as supporting active learning principles by providing authentic, emotionally engaging scenarios that trigger reflection [5, 7]. Educators described the VR experience as more realistic than traditional role-play, which resonates with research emphasizing VR’s potential to enhance authenticity and learner engagement [23, 33, 43, 44]. Similarly, students reported that the immersive nature of the VR scenarios contributed to a stronger emotional engagement and a more embodied sense of experiencing challenging interactions. Many appreciated the safe environment created by small, familiar groups, which helped them manage vulnerability and stress. These comments reinforce the importance of designing VR activities that balance emotional challenges with psychological safety. However, students and educators attributed the emotional immediacy, sense of presence, and ability to interact with a child avatar specifically to the VR modality, whereas the active learning outcomes (peer learning, feedback exchange, reflective dialogue) were linked to the simulation pedagogy rather than the technology itself. Importantly, VR simulation enabled the inclusion of a child avatar, which participants experienced as adding a level of realism and interaction that can be difficult to achieve in standard peer role-play, adding complexity and realism to the interaction. Participants perceived the child avatar and immersive environment as features that differentiated VR from traditional role‑play, which may indicate potential pedagogical advantages [45]. Recent reviews confirm that VR integration in medical education is expanding rapidly, with evidence pointing to its effectiveness for both technical and non-technical skills, including communication and teamwork [46].

However, it is worth questioning whether the observed learning outcomes stemmed primarily from the simulation itself, the active learning principles embedded in the design, or a combination of both. The structured integration of prebriefing, simulation, and debriefing, combined with collaborative group work, likely amplified the educational impact. This interplay reflects Biggs’ concept of constructive alignment, where learning activities, assessment, and intended outcomes are coherently linked [13]. In this study, VR functioned primarily as a trigger for engagement, while the learning gains were rooted in the reflective processes embedded in the simulation design which was explicitly aligned with learning objectives focused on open-ended questioning and family-centered communication, and the debriefing phase reinforced these objectives through guided reflection [47, 48]. Our findings therefore suggest that the perceived educational benefits were mainly associated with established principles of simulation-based education, such as structured reflection, feedback, and facilitation, while the VR component contributed by enhancing engagement and experiential realism rather than acting as the primary driver of learning. The present study cannot disentangle these effects empirically, but participant reports indicate that the VR component primarily served as an immersive trigger for reflection, whereas the perceived learning processes were linked to the broader simulation pedagogy.

### The role of reflection, feedback, and feedforward

A key insight from this study is the central role of the debriefing phase. Both students and educators emphasized that the most substantial learning emerged during the debriefing phase, where structured guidance enabled students to process their experiences and compare different approaches. These reflective processes are inherent to simulation-based education. They appreciated having time to discuss strategies, share observations, and receive feedback from peers and facilitators. One student noted that they learned how to involve everyone in the conversation, while another valued observing peers being vulnerable in front of each other. The VR scenarios contributed by introducing emotionally charged material for reflection, but it was the debriefing that supported the development of communication skills, feedback literacy, and professional reasoning. This further supports the interpretation that the most meaningful learning processes were linked to the pedagogical structure of the simulation, particularly the debriefing phase, rather than to the VR technology itself. These insights align with literature suggesting that collaborative reflection can foster metacognitive development and confidence [15], although our data captures only participants’ perceptions of learning.

Reflection during debriefing is widely recognized in the literature as the cornerstone of simulation-based learning, enabling students to consolidate experiences and develop clinical reasoning. In our study, participants also described the debriefing as the phase where they perceived the most meaningful learning. Structured debriefing models such as Promoting Excellence and Reflective Learning in Simulation (PEARLS) [49] and the International Nursing Association for Clinical Simulation and Learning (INACSL) Standards of Best Practice [14] emphasize guided reflection combined with feedback and feedforward to optimize learning outcomes. Feedback provides learners with insight into current performance, while feedforward focuses on future improvement, creating a continuous learning cycle [50]. Previous studies show that facilitator actions, such as asking open-ended questions and encouraging self-assessment, trigger deeper reflection and critical thinking [51]. Innovative approaches, like reflective learning conversations, further integrate Bloom’s taxonomy and appreciative inquiry to enhance metacognitive engagement [52]. Collectively, these findings underscore the literature describing debriefing as a structured, dialogic process that supports the interpretation of experience. In our study, participants perceived this phase as particularly meaningful for their learning.

In addition to the stated objectives, the study revealed several implicit or “hidden” learning outcomes. Students developed the courage to try and fail, emotional resilience, the ability to tolerate uncertainty, and the capacity to give and receive constructive feedback. Educators also noted that students gained confidence not only in their communication skills but in navigating complex interpersonal dynamics, skills that are essential in real clinical settings but often difficult to cultivate through traditional lectures. These outcomes emerged organically through the combination of immersive VR scenarios and structured reflection, suggesting that educational design should intentionally recognize and support such implicit learning processes. This aligns with transformative learning theory, which emphasizes shifts in perspective through critical reflection [15], and underscores VR’s potential to foster professional growth beyond technical skills. However, although our data highlights that students and educators perceived the debriefing phase as the most meaningful component, we cannot conclude that debriefing led to improved measurable learning outcomes. Instead, our findings align with established simulation literature that positions structured reflection as a central pedagogical mechanism [14, 49].

### Technical and pedagogical preconditions

Successful implementation of VR simulations depended on reliable technical support and clear logistics. Educators expressed anxiety about potential technical failures and emphasized the need for “super-users” and contingency plans. These findings echo previous research on the importance of technical reliability in simulation-based education [18, 46]. Students also commented on technical limitations, noting that avatar responses sometimes felt unnatural, and that short scenarios limited their ability to practice communication skills. These concerns suggest that technology design and scenario length are critical factors for perceived learning quality [43].

Beyond technical stability, pedagogical structure and facilitator competence played a decisive role. Educators highlighted that having a detailed facilitation guide and clear learning outcomes reduced stress and improved their confidence in managing the sessions. This structured approach aligns with best practices for simulation-based education, where prebriefing, scenario execution, and debriefing are carefully planned to ensure constructive alignment [13, 14]. Educators noted that without this structure, students might feel exposed or uncertain, which could undermine psychological safety, a prerequisite for effective learning.

The findings also underscore that reflection is most effective when guided by a trained facilitator. Educators emphasized that structured pedagogical models and clear learning outcomes were crucial for creating a psychologically safe environment. Students confirmed this, expressing that facilitator guidance during debriefing helped them feel “seen and heard,” and that structured feedback made the experience less intimidating. Unguided simulations risk leaving students without the necessary support to process complex experiences, which might potentially limit learning. This observation aligns with evidence that instructional strategies involving dialogue and educator-led questioning significantly improve critical thinking skills [5] and are reinforced by VR’s immersive potential [48].

Another critical insight was the role of facilitator training. Educators who had completed a basic facilitation course reported feeling more competent in guiding reflections and applying feedback/feedforward principles. This suggests that investing in facilitator development is essential for scaling VR-based learning. In addition, the collaborative development and delivery of the simulations across the physiotherapy and occupational therapy programs were perceived as supporting facilitation quality, by enabling shared preparation, exchange of pedagogical approaches, and mutual support among educators. Interdisciplinary collaboration among facilitators was perceived as enriching and resource-efficient, allowing educators to share expertise and reduce the burden of preparation while maintaining a consistent pedagogical approach.

Finally, resource allocation emerged as a practical challenge. VR simulations require more time and personnel compared to traditional lectures. Educators proposed strategies such as reducing lecture time in favor of self-study and reallocating resources to student-active learning formats. Although these preconditions were discussed in relation to VR simulation, they reflect fundamental requirements for simulation‑based teaching and learning using educational technologies more broadly, rather than being specific to VR alone. These considerations highlight that successful integration of VR into curricula is not only a technological endeavor but also a systemic pedagogical shift requiring institutional commitment.

### Limitations

This study contains some limitations that need to be considered. First, less than half of the students responded to the questionnaire, which may introduce response bias and limit the generalizability of the findings. Additionally, the study utilized a self-developed questionnaire that was not formally validated prior to use. While this allowed us to capture context-specific aspects of the VR simulation experience, the lack of established validity and reliability represents a limitation. Second, student feedback revealed mixed views on whether VR added significant value compared to traditional role-play. While some questioned its necessity, others emphasized that VR enabled inclusion of a child avatar and created emotional engagement, advantages that are not achievable through peer-based simulations. Third, the VR scenarios were brief, and avatar responses occasionally misaligned with student questions, reducing perceived realism. These technological constraints may have affected the depth of communication practice. Fourth, the number of educators included in the focus group was limited, and data saturation cannot be assumed. While the participants had direct experience with the implementation, this may limit the breadth of perspectives captured. Additionally, the study primarily captures students’ and educators’ perceptions rather than objectively measured learning outcomes. Therefore, conclusions about the effectiveness of VR or debriefing should be interpreted with caution. Our findings reflect how participants experienced the VR simulations and the surrounding pedagogical structure, but they cannot establish whether VR improved communication skills or whether debriefing was the definitive mechanism responsible for learning. The study also cannot disentangle which effects were specific to the VR technology and which were attributable to general principles of simulation-based education (e.g., structured facilitation, feedback, and reflective dialogue).

### Implications for practice and research

The findings have several implications for health professional educations. First, VR simulation appears to be an effective environment for developing communication skills in challenging clinical dialogues, particularly when combined with structured reflection and guided facilitation. This highlights the need to integrate technology-enhanced learning methods into curricula, not as a replacement for clinical practice, but as a preparatory tool that allows students to rehearse in a safe environment. Student satisfaction, which emerged as a strong indicator of quality in this study, reinforces the value of such approaches. Satisfaction was linked to factors such as group harmony, facilitator support, and case authenticity, all of which are associated with deeper engagement and improved learning outcomes [44]. Generally, VR should be understood not as a standalone method for communication training, but as a complementary tool that can enrich simulation-based education by providing realistic, emotionally engaging scenarios that feed into deeper reflective learning. Second, institutions must invest in technical infrastructure and facilitator training to ensure smooth implementation and psychological safety. Reliable technology prevents disruptions that can undermine immersion and learning, while trained facilitators create a safe environment for risk-taking and guide structured debriefing using feedback and feedforward principles. Evidence-based frameworks such as PEARLS and INACSL standards show that facilitator competence directly impacts learning outcomes. Together, these investments ensure that VR simulation is both technically stable and pedagogically effective. Third, future research should explore how VR can be optimized to train complex communication skills and examine the impact of varying degrees of interactivity on learning outcomes. Longitudinal studies could also investigate whether skills acquired through VR simulations transfer effectively to clinical practice.

## Conclusion

This study contributes to the growing evidence that VR-based simulations may serve as a valuable pedagogical tool in healthcare education. Participants described the VR scenarios as immersive and emotionally engaging and perceived the structured debriefing as the most meaningful part of the learning process. By offering students a safe, immersive, and emotionally engaging environment to practice communication skills, VR simulations were perceived as supporting both explicit and implicit learning processes. Importantly, the educational value of VR appeared not to lie in the technology alone, but in how it was embedded within a structured simulation of pedagogy. The most meaningful learning was associated with the debriefing process, where reflection, feedback, and guided facilitation supported the development of communication skills and professional reasoning. In this sense, VR functioned primarily as a catalyst for engagement and reflection within a broader simulation framework. The structured integration of prebriefing, simulation, and debriefing, combined with facilitation and technical support, was essential for maximizing the educational value of VR. These findings highlight that successful implementation depends not only on technological innovation, but on thoughtful pedagogical design. As healthcare education continues to evolve, incorporating innovative, student-centered approaches like VR simulation may play a key role in preparing future professionals for the complex interpersonal challenges of clinical practice. As advances in educational technologies accelerate, there will be a growing need to continually refine the pedagogical approaches, facilitation practices, and curricular models that accompany them, ensuring that technological innovation is matched with equally thoughtful educational design.

## Supplementary Information

Supplementary Material 1.

Supplementary Material 2.

## Data Availability

Due to the nature of this research, participants did not consent to their full, raw interview data being shared publicly. Supporting data are available from the corresponding author upon reasonable request.
