# Supplementary material for: Use of virtual reality simulation in preparation for practical training in healthcare education – a mixed method study with students and educators
Source: BMC Med Educ. 2026 May 30;26:1237. doi: 10.1186/s12909-026-09579-9 (PMC13435449; doi:10.1186/s12909-026-09579-9)
Supplement: Supplementary file 1 [file 12909_2026_9579_MOESM1_ESM.docx]

# Supplementary material 1

## Student questionnaire

1. I liked simulation as a learning method.
2. I believe simulation can be an important learning activity for developing my communication skills.
3. I felt that my prior knowledge was sufficient to carry out the dialogue.
4. I experienced that the simulation provided opportunities to achieve learning outcomes in communication.
5. I gained professional/academic benefit from being an active participant in the simulation (the one wearing the VR headset).
6. I gained professional/academic benefit from being a respondent while other group members completed the simulation.
7. The reflections afterwards gave me the opportunity to think about what happened during the simulation.
8. The reflections afterwards reassured me about what I mastered well.
9. The reflections afterwards gave me the opportunity to learn what I could have done differently.
10. I feel that I learned a lot from my fellow students during the reflection sessions.
11. The teachers were engaged, provided constructive feedback, and contributed to learning.
12. I felt safe in my group.
13. After the session, I left the room with a sense of mastery/accomplishment.
14. After the simulation, I feel more confident ahead of clinical practice.
15. After the simulation, I feel more confident that I have the basic skills needed to meet patients and relatives in clinical practice.
16. I believe simulation should be used as part of my education.
17. Please name up to three aspects that worked well in the simulation exercise.
18. Please name up to three aspects that could have been done differently in the simulation exercise.
