# Supplementary material for: Use of virtual reality simulation in preparation for practical training in healthcare education – a mixed method study with students and educators
Source: BMC Med Educ. 2026 May 30;26:1237. doi: 10.1186/s12909-026-09579-9 (PMC13435449; doi:10.1186/s12909-026-09579-9)
Supplement: Supplementary file 2 [file 12909_2026_9579_MOESM2_ESM.docx]

# Supplementary material 2

## Interview guide

Through a semi-structured focus group interview, we aim to explore educators’ experiences (both positive and negative) with the use of VR-based dialogue simulation with second-year physiotherapy and occupational therapy students. The interview focuses on the following themes: (a) the practical implementation of the VR simulation; (b) students’ learning outcomes from the VR simulation; (c) the facilitators’ own learning experiences; (d) collaboration between the physiotherapy and occupational therapy programs; and (e) other experiences that the educators consider important to share.

**Interview questions:**

- We would like to start by asking you to describe your experiences with conducting the VR-based dialogue simulation, can you please tell us about your experiences?
  – How did the practical organization work (oral and written information, practical implementation and support in using the VR equipment, sequencing of cases)?
  – How did you experience the facilitator guide/template?
- How do you think the students experienced participating in the VR simulation?
  – How did the dialogue simulation itself work in practice?
  – How did the post-simulation reflections work?
  – What do you think the students took away from / learned by participating?
  – What are your thoughts on the number of students in each group?
- Can you tell us about what you feel you personally learned from acting as a facilitator in the VR-based dialogue simulation?
- How did you experience the collaboration between the study programs?
  – Is there anything you think should have been done differently?
  – How do you envision this collaboration being continued in the future?
- Are there any other experiences you consider important to share with us?
- What do you think are the most important take-home messages from your experiences?
  – What do you think is important to carry forward?
  – What do you think could have been done differently?
